# Pattern recognition receptors in health and diseases

**DOI:** 10.1038/s41392-021-00687-0

**Published:** 2021-08-04

**Authors:** Danyang Li, Minghua Wu

**Affiliations:** 1 Hunan Provincial Tumor Hospital and the Affiliated Tumor Hospital of Xiangya Medical School, Central South University, Changsha, Hunan China; 2 The Key Laboratory of Carcinogenesis of the Chinese Ministry of Health, The Key Laboratory of Carcinogenesis and Cancer Invasion of the Chinese Ministry of Education, Cancer Research Institute, Central South University, Changsha, Hunan China

**Keywords:** Drug development, Innate immunity, Structural biology

## Abstract

Pattern recognition receptors (PRRs) are a class of receptors that can directly recognize the specific molecular structures on the surface of pathogens, apoptotic host cells, and damaged senescent cells. PRRs bridge nonspecific immunity and specific immunity. Through the recognition and binding of ligands, PRRs can produce nonspecific anti-infection, antitumor, and other immunoprotective effects. Most PRRs in the innate immune system of vertebrates can be classified into the following five types based on protein domain homology: Toll-like receptors (TLRs), nucleotide oligomerization domain (NOD)-like receptors (NLRs), retinoic acid-inducible gene-I (RIG-I)-like receptors (RLRs), C-type lectin receptors (CLRs), and absent in melanoma-2 (AIM2)-like receptors (ALRs). PRRs are basically composed of ligand recognition domains, intermediate domains, and effector domains. PRRs recognize and bind their respective ligands and recruit adaptor molecules with the same structure through their effector domains, initiating downstream signaling pathways to exert effects. In recent years, the increased researches on the recognition and binding of PRRs and their ligands have greatly promoted the understanding of different PRRs signaling pathways and provided ideas for the treatment of immune-related diseases and even tumors. This review describes in detail the history, the structural characteristics, ligand recognition mechanism, the signaling pathway, the related disease, new drugs in clinical trials and clinical therapy of different types of PRRs, and discusses the significance of the research on pattern recognition mechanism for the treatment of PRR-related diseases.

## Introduction

The first line of defense against pathogens that gradually evolved in organisms is innate immunity,^1^ which is divided into two levels: first, the skin, mucosal tissue, blood–brain barrier, and chemical barrier (e.g. fatty acid, pH, enzyme, and complement system) of the host can effectively resist the invasion of general pathogenic microorganisms;^2–4^ second, the innate immune system of vertebrates protects the organism through nonspecific immune defense and surveillance by innate immune cells. Innate immune cells mainly include monocytes, neutrophils, macrophages, dendritic cells, natural killer (NK) cells, mast cells, eosinophils, and basophils.^5,6^ Unlike T cells and B cells, which have high specificity, innate immune cells do not express specific antigen recognition receptors. Through the recognition and binding of some common molecules on the surface of pathogens, apoptotic host cells, and damaged senescent cells, pattern recognition receptors (PRRs) induce immunoprotective effects, such as anti-infection and antitumor effects, and participate in the initiation and effect process of specific immune response.^7–9^

In the 1990s, the hypothesis of pathogen-associated molecular patterns (PAMPs) and PRRs that recognize PAMPs was proposed by Janeway, which was of epoch-making significance and changed research on innate immunity.^10^ The main point of this hypothesis is the connection between the innate immune signal and the initiation of the adaptive immune response. Some unique and conserved components of pathogenic microorganisms can induce the second signal required to activate T cells, so as to control the adaptive immunity from being activated under normal conditions.^11,12^ In addition, there are a class of receptors in the host that can recognize pathogenic microorganisms and activate the second signal in time, which are independent of gene rearrangement. In vertebrates, innate immunity recognizes pathogenic microorganisms and assists in the activation and expression of second signals that activate the adaptive immunity.^13^

Toll-like receptors (TLRs) are one of the earliest PRRs discovered in the innate immune system, which plays an important role in inflammatory responses.^14,15^ Therefore, here is a brief description of the development history of PRRs with TLRs as a representative. TLRs were first found in Drosophila in the form of genes in 1994. Studies have shown that the function of this gene is related to the formation of the dorsal–ventral axis during the embryonic development of Drosophila.^16^ In 1988, Hashimoto et al. discovered that the Toll gene encodes a transmembrane protein and clarified the structure of the Toll protein.^17^ In 1991, Gay et al. found that Toll protein had structural homology with interleukin-1 (IL-1), a natural immune molecule in mammals, suggesting that the function of Toll may be related to immunity.^18^ In 1996, Hoffmann team found that Toll plays a role in the resistance of Drosophila to fungal infection. Toll-activated mutants persistently express antifungal peptides, while Toll-deletion mutants, on the contrary, lose their ability to arrest fungal infection. It has been found that Toll can recognize spatzle (an important protein in the development of the dorsal and abdomen of Drosophila) and initiate a series of signal transduction to activate the expression of antifungal peptide.^19^ In 1997, Janeway et al. cloned human TLR4. TLR4 can induce the activation of nuclear factor (NF)-κB and the expression of the co-stimulatory molecule CD80. This proves that innate immunity recognizes pathogenic microorganisms and activates the expression of the second signal, which is indispensable for the activation of adaptive immunity.^20^ Since the discovery of TLR4, many PRRs and their corresponding ligands have been discovered. PRRs can be divided into the following five types based on protein domain homology: TLRs, nucleotide oligomerization domain (NOD)-like receptors (NLRs), retinoic acid-inducible gene-I (RIG-I)-like receptors (RLRs), C-type lectin receptors (CLRs), and absent in melanoma-2 (AIM2)-like receptors (ALRs) (Table 1).^21^ PRRs are representative of immune receptors in innate immunity and exist in various forms. PRRs are not only expressed on the cell membrane but also widely distributed in intracellular compartment membranes and the cytoplasm.^22^ Membrane-bound PRRs and PRRs in the cytoplasm are basically composed of ligand recognition domains, intermediate domains, and effector domains.^23,24^ PRRs activate downstream signaling pathways through recognition of their ligands. The activation of downstream signaling pathways can produce many effects: recruiting and releasing cytokines, chemokines, hormones, and growth factors; inducing chronic inflammation; forming an inflammatory microenvironment; initiating innate immune killing and subsequent acquired immune response,^9^ maintaining the balance of host microecology; and eliminating dead or mutated cells.

**Table 1:** Common PRRs in innate immunity

Items	PRR	Domains	Cellular distribution	PAMP	Sources	Signaling pathways
Toll-like receptors (TLRs)	TLR1 (TLR1–TLR2)	LRR domain–transmembrane domain–TIR domain (extracellular to intracellular)	Mo, DC, Ma, Eo, Ba	Triacyl lipopeptide	Bacteria	Most TLRs: MyD88-dependent pathways; TLR3: TRIF-dependent pathways; TLR4: MyD88-dependent pathways and TRIF-dependent pathways
	TLR2 (TLR1–TLR2, TLR2–TLR6)		Mo, DC, Ma, Eo, Ba	Lipoteichoic acid	Bacteria	
				Arabinomannan	Mycobacterium	
				Peptidoglycan	Bacteria	
				Zymosan	Fungi	
				Lipoprotein	Mycoplasma	
				Pore protein	Neisseria	
	TLR3		Mφ, DC, IEC	dsRNA	Virus	
	TLR4 (MD-2/CD14)		Mφ, DC, Ma, Eo	Lipopolysaccharides	Bacteria	
				Heat-shock proteins	Host	
	TLR5		IEC	Flagellin	Bacteria	
	TLR6 (TLR2–TLR6)		Mo, DC, Ma, Eo, Ba	Lipoteichoic acid	Bacteria	
				Peptidoglycan	Bacteria	
	TLR7		pDC, Mφ, Eo	ssRNA	Virus	
				Imidazoquinoline	Artificially synthesized	
	TLR8		Mφ, N	ssRNA	Virus	
	TLR9		pDC, Eo, Ba	Non-methylated CpG DNA	Bacteria, Virus	
	TLR10 (human)		pDC, Eo, Ba	dsRNA	Virus	
	TLR11 (mouse)		Mφ, DC	Profilin and related proteins	*Toxoplasma gondii*	
	TLR12 (mouse)		DC	Profilin and related proteins	*Toxoplasma gondii*	
	TLR13 (mouse)		Unknown	23s ribosomal RNA	Bacteria	
Nucleotide-binding oligomerization domain-like receptors (NLRs)	NOD1	LRR domain–NBD–effector domains	IEC, cytosol of Mφ	iE-DAP	Gram negative bacteria	RIP2-TAK1-NF-κB pathways
	NOD2			MDP	Gram-negative bacteria, Gram-positive bacteria	
RIG-I-like receptors (RLRs)	RIG-I	(RD)-CTD-DexD/H helicase domain–CARD	Cytosol	5’-triphosphorylated RNA, short-chain dsRNA	Virus	MAVS-TRAF6-NF-κB/TBK1 pathways
	MDA5			poly IC, long-chain dsRNA	Virus	
	LGP2			dsRNA	Virus	
C-type lectin receptors (CLRs)	Dectin-1	CTLD–ITAM	DC, Mφ	β-Glucan	Fungus	Tyrosine kinase-dependent and non-tyrosine kinase-dependent pathways
	Dectin-2			α-Mannan	Fungus	
Absent in melanoma-2-like receptors (ALRs)	ALRs	HIN-200-PYD	Cytosol	dsDNA	Bacteria	Inflammasome–pyroptosis

*LRR* leucine-rich repeat, *TIR* Toll/IL-1R domain, *NBD* nucleotide-binding domain, *RD* repressor domain, *CTD* C-terminal domain, *CARD* caspase activation and recruitment domain, *CTLD* C-type lectin-like domains, *ITAM* immunoreceptor tyrosine-based activation motif, *PYD* pyrin domain, *Mo* monocyte, *DC* dendritic cell, *Ma* mastocyte, *Eo* eosinophils, *Ba* basophils, *pDC* plasmacytoid dendritic cell, *IEC* intestinal epithelial cell, *N* neutrophil, *dsRNA* double-stranded RNA, *ssRNA* single-stranded RNA, *iE-DAP* γ-D-glu-meso-diaminopimelic acid, *MDP* muramyl dipeptide, *MyD88* myeloid differentiation factor 88, *TRIF* TIR domain-containing adaptor protein-inducing interferon β, *RIP2* receptor-interacting serine–threonine protein 2, *TAK1* transforming growth factor-β-activated kinase 1, *NF-κB* nuclear factor κB, *MAVS* mitochondrial antiviral signaling protein, *TRAF6* tumor necrosis factor receptor-associated factor, *TBK1* TANK-binding kinase 1

PAMPs are the specific and highly conserved molecular structures shared by the same kind of pathogenic microorganisms,^25,26^ including lipids, proteins, and nucleic acids, such as lipopolysaccharides (LPS), lipoteichoic acid (LTA), and bacterial DNA.^27,28^ PAMPs are essential for pathogen survival and usually have unique molecular or subcellular characteristics that are not found in host cells. Therefore, innate immune cells can recognize PAMPs via PRRs, distinguish “self” and “non-self,”^29^ and respond to pathogens and their products. However, the host will produce some proteins and metabolites after being stimulated by its own tissue damage, cell necrosis, and other factors.^30^ These molecules are called damage-associated molecular pattern (DAMP).^31^ PRRs can also recognize such molecules, activate natural immunity, and cause inflammation.^32^

With advances in research on PRRs structure and distribution at different levels, the role of PRRs in the innate immune regulatory network has become clearer. The recognition and binding of PRRs to their ligands is critical in initiating the innate immune response. Therefore, the study of PRR-mediated pattern recognition mechanisms will help to elucidate the signaling pathways and mechanisms of disease and provide new targets and methods for the treatment of diseases. In this review, we describe the structural characteristics, ligand recognition mechanism, the signaling pathway, the related disease, new drugs in clinical trials, and clinical therapy of different types of PRRs in detail. We focus on the different domains and ligand recognition mechanisms between PRRs, which can not only provide new ideas for the definition, role, and clinical application of PRRs but also promote the study of the role of the innate immune system in related diseases and even tumors.

## PRRs and ligand-recognition mechanisms

### Toll-like receptors

**Fig. 1:**
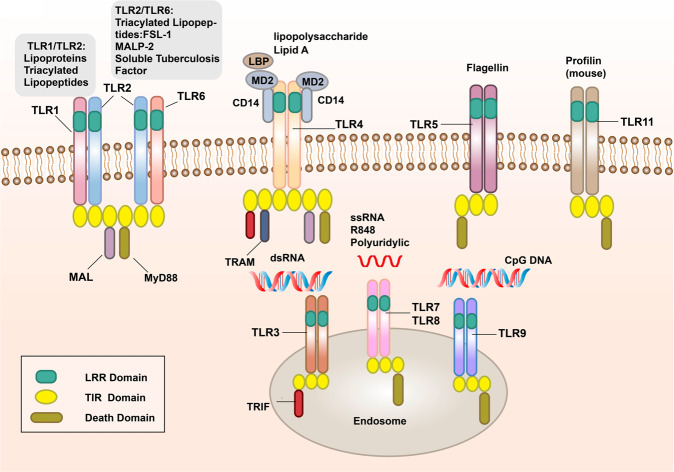
The signal transduction pathways and structure of TLR-binding ligand complex. TLRs can recognize one or more PAMPs through LRR domain. They usually dimerize themselves and recruit adaptor molecules with the same TIR domain to transmit signals

TLRs are membrane-bound signal receptors and are important PRRs in the innate immune system of vertebrates.^15,33^ Such receptor molecules usually have two functions, one is to bind specifically to the ligand, and the other is to transmit signals. The corresponding signal transduction will amplify the effect of anti-pathogen infection, so that the immune cells active in the inflammatory response can be activated through the transcription of genes, and produce and secrete a variety of pro-inflammatory and antiviral factors.^34–36^ Up to now, 10 functional TLRs (TLR1–10) have been found in humans and 12 (TLR1–9 and TLR11–13) in mice.^37–41^ TLR10 in mice is not functional due to the insertion of reverse transcriptase.^42^ TLRs recognize PAMPs in different subcellular structures. The cellular localization of TLRs determines the types of ligands and the recognition mechanism. Some TLRs (TLR1, 2, 4, 5, 6, 10) are expressed on the surface of immune cells in the form of heterodimers or homodimers, mainly recognizing the membrane components of pathogenic microorganisms, such as lipids, lipoproteins, and proteins; others (TLR3, 7, 8, 9) are expressed in the form of homodimers, which mainly recognize the nucleic acids of microorganisms (Fig. 1).^43^

TLRs are type I transmembrane glycoproteins and are composed of an extracellular region, a transmembrane region, and an intracellular region.^44^ The extracellular region contains leucine-rich repeats (LRRs), which are responsible for the recognition of specific ligands and perform extracellular pattern recognition. The intracellular domain contains the same Toll/IL-1R (TIR) domain as IL-1R, which plays a role in signal transduction. The extracellular region of TLRs contains LRRs, which mediate the pattern recognition of TLRs (Fig. 1).^45^ In 2007, researchers used X-ray crystal diffraction to analyze and determine the structure of the TLR–ligand complex,^46^ which provided a deeper understanding of the LRR domain. The LRR domain is shaped like a horseshoe, and each module consists of a conserved leucine motif and a variable region. The “LxxLxLxxN (L leucine, x any amino acid, N asparagine)” motif is composed of 20–30 amino acids and is on the concave surface of the horseshoe-like structure.^47–50^ The horseshoe-shaped N-terminus and C-terminus contain disulfide bridges formed by cysteine clusters^51,52^ to protect the hydrophobic core. After TLRs recognize and bind the corresponding PAMPs and endogenous ligands, the TIR domains conduct signals by binding to different receptor adaptor proteins in the cytoplasmic region.^53,54^ The TIR domain has three conserved amino acid sequences, which are called 1,2,3 cassettes. Depending on the different adaptor proteins, TLRs signaling can be divided into myeloid differentiation factor 88 (MyD88)-dependent and MyD88-independent pathways (Fig. 1).^55,56^

**Fig. 2:**
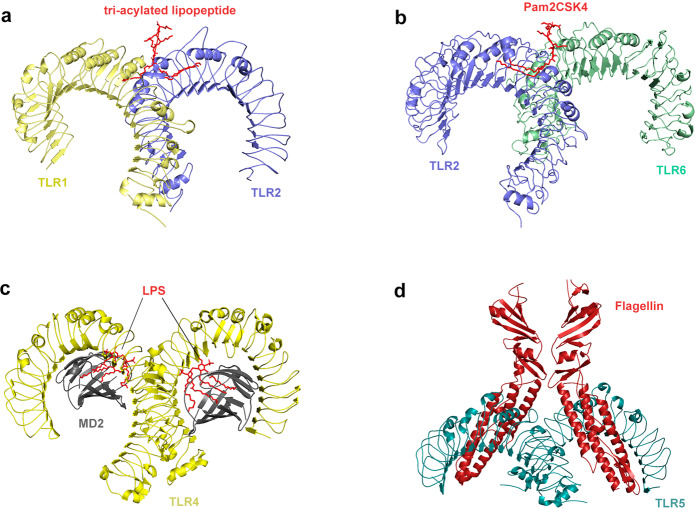
Crystal structure of TLRs with ligands. **a** Crystal structure of the TLR1–TLR2 heterodimer induced by binding of a tri-acylated lipopeptide (PDB 2Z7X). TLR2 initiates immune responses by recognizing di-acylated and tri-acylated lipopeptides. The ligand specificity of TLR2 is controlled by whether it heterodimerizes with TLR1 or TLR6. Binding of the tri-acylated lipopeptide (red) induced the formation of M-type crystal structures of the TLR1 (pale yellow) and TLR2 (slate) ectodomains. **b** Crystal structure of TLR2–TLR6–Pam2CSK4 complex (PDB 3A79). Binding of the di-acylated lipopeptide, Pam2CSK4 (red), induced the formation of M-type crystal structures of the TLR2 (slate) and TLR6 (pale green) ectodomains. **c** Crystal structure of mouse TLR4/MD2/LPS complex (PDB 3VQ2). After LPS (red) binds with the TLR4 (yellow)/MD2 (gray) complex, the hydrophobic pocket of MD2 is used to bridge the two TLR4–MD2–LPS complexes to form a spatially symmetrical M-type structure. Mouse TLR4/MD2/LPS exhibited an complex similar to the human TLR4/MD2/LPS complex. **d** Crystal structure of the N-terminal fragment of zebrafish TLR5 in complex with Salmonella flagellin (PDB 3V47). Two TLR5 (cyan)–flagellin (firebrick) 1:1 heterodimers assemble into a 2:2 tail-to-tail signaling complex to function

Exploring the pattern recognition mechanisms of TLRs is very valuable for understanding innate immunity and some tumorigenesis mechanisms. Therefore, researchers used X-ray crystal diffraction to determine the crystallographic structure of the extracellular domain of TLRs and the ligand complex. Although the ligand complexes have different structures, all these complexes have similar M-type crystal structures (Fig. 2).^50,51^ TLR1 or TLR6 can form TLR1/TLR2 and TLR6/TLR2 heterodimers with TLR2 to recognize tri-acylated lipopeptide and di-acylated lipopeptide,^57^ respectively. After recognizing the appropriate ligands, TLR2 can form an M-type structure with the extracellular region of TLR1 and TLR6, and the pocket structure formed binds to the ligand.^58–61^ The crystal structure of TLR1–TLR2–tri-acylated lipopeptide complex is similar to that of TLR2–TLR6–di-acylated lipopeptide complex, but there are important structural differences between TLR1 and TLR6 in the ligand-binding site and dimerization surface. The ligand-binding pocket of TLR1–TLR2 is located in the interface between the central and C-terminal domain, and TLR1–TLR2–tri-acylated lipopeptide is stabilized by non-covalent bonds such as hydrogen bonds, hydrophobic interactions, and ionic interactions near the ligand-binding pocket.^62^ In TLR6, the side chains of amino acid residues block the ligand-binding pocket, resulting in a pocket less than half the length of TLR1. In addition, the TLR2–TLR6 heterodimer is mainly regulated by the surface exposed residues of the LRR11–14 module (Fig. 2a, b).^63^ Researches on the structure of ligand complexes can significantly promote the discovery of small molecule agonists/antagonists targeting PRRs. A recent study revealed the activation mechanism of atypical agonists for TLR1–TLR2. Diprovocim is a recently found small molecule activator for TLR1–TLR2, but it has no structural similarity with the tri-acylated lipopeptide complex. It also interacts with TLR1–TLR2 in the same binding pocket as typical lipopeptide ligand.^64^ Crystal structure analysis revealed that double-stranded RNA (dsRNA) binds to the LRR domains of the N-terminus and C-terminus of TLR3.^65,66^ Different from the way that other TLRs directly recognize ligands,^67–69^ TLR4 specifically recognizes LPS in combination with two auxiliary molecules, myeloid differentiation factor 2 (MD2) and the LRR structural protein CD14. LPS is transported by LPS-binding protein to CD14 on the cell membrane of monocytes and macrophages to form a complex and then interacts with TLR4/MD2.^70^ After LPS binds with the TLR4/MD2 complex, the hydrophobic pocket of MD2 is used to bridge the two TLR4–MD2–LPS complexes to form a spatially symmetrical M-type TLR4–MD2–LPS dimer,^71^ and then conformational changes affect their respective functional domains and transmit signals (Fig. 2c). In addition to binding to LPS, TLR4 is also involved in the recognition of natural products (carnosic acid, paclitaxel) and pneumolysin.^72–74^ TLR5 is the most conserved and important PRR, which is usually stimulated by bacterial flagellin. In the form of homodimer, TLR5 plays a major role in the primary defense of invasive pathogens and immune homeostasis regulation.^75^ Although the heterodimeric structure of TLR5a–TLR5b in zebrafish and the crystallographic structure of TLR5–flagellin complex has been clearly reported, the lack of biochemical and structural information of fish TLR hinders the understanding of flagellin-based therapies. In the future, experimental TLR5–flagellin complex structure modeling and computational simulation should be used to study flagellin-mediated interactions between various pathogens and host immune receptors (Fig. 2d).^76^ It has been reported that TLR1–6 each exist as monomers in solution, and dimerization occurs only when the ligand is bound; in contrast, TLR8 and TLR9 exist as preformed dimers, and the binding of ligands induces conformational changes in preformed dimers (Fig. 1).^49,77,78^ Lee revealed that TLR10 binds dsRNA in vitro at endosomal pH, indicating that dsRNA is a ligand of TLR10. The recognition of dsRNA by TLR10 recruits MyD88, thereby transducing signals and inhibiting interferon regulatory factor 7 (IRF7)-dependent type I interferon (IFN) production.^79^ In mice, TLR11 and TLR12 are the main effector molecules to recognize *Toxoplasma gondii*. The recognition of *T. gondii* profilin by TLR11 depends on the parasite-specific, surface-exposed motif in TgPRF consisting of an acidic loop and a β-hairpin.^80–82^

### NOD-like receptors

**Fig. 3:**
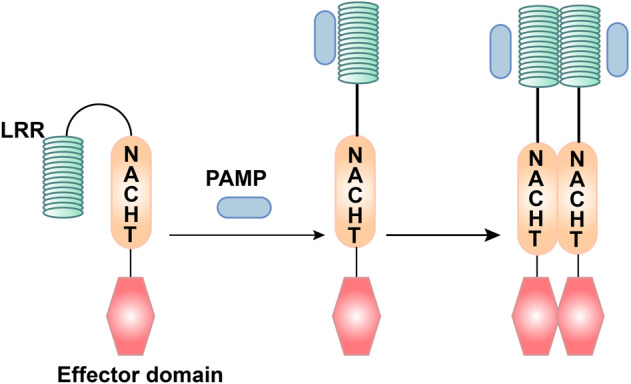
The ligand recognition mechanism of NLRs. The combination of PAMP and LRR changes the conformation of NLRs from self-inhibition to activation

The growth cycle of some pathogenic microorganisms involves infection of the cytoplasm. For example, viral genes are often transcribed and translated in the cytoplasm, and virus particles are assembled. In addition, some bacteria and parasites have a series of escape mechanisms, such as making holes in the phagosome membrane and entering the cytoplasm. Therefore, pathogens and their components, as well as other components produced by infection and injury, will appear in the cytoplasm,^83^ which requires the recognition of PRRs in the body. NLRs are intracellular PRRs, composed of three domains:^84,85^ one is the central nucleotide-binding domain (NBD), also known as the NACHT domain (synthesized by the abbreviations of the following four kinds of NLR members: NAIP, CIITA, HETE, TP1), which is shared by the NLR family and is very important for nucleic acid binding and oligomerization of NLRs (Fig. 3); LRRs at the C-terminus, which are used to identify ligands; and the N-terminal effector domain, which is the protein interaction domain, such as the caspase activation and recruitment domain (CARD) or the pyrin domain (PYD).^86–89^ According to the different N-terminal effector domains, the NLRs family can be divided into five subfamilies: the NLRC subfamily, which contain CARDs; the NLRP subfamily, which contain PYDs; the NLRB subfamily, which contain baculovirus inhibitor of apoptosis protein repeats; the NLRA subfamily, which contain acidic activation domains; and the NLRX subfamily containing other NLR effector domains.^85^

Among the NLRs family, the most in-depth study has focused on NOD1 and NOD2 proteins. NOD1 mainly recognizes the diaminopimelic acid (γ-D-glu-meso-diaminopimelic acid (iE-DAP)) of the cell wall of Gram-negative bacteria.^90,91^ In addition to recognizing muramyl dipeptide (MDP) in all bacterial cell walls, NOD2 can also recognize single-stranded RNA (ssRNA) of the virus, but it must be a complete viral ssRNA.^92^ The basic process of NOD2 activation and signal transduction is as follows: after pathogenic bacteria are phagocytosed by macrophages, they first form phagosomes, and then fuse with lysosomes to become phagolysosomes. Under the action of lysosomal enzymes, bacterial cell wall components are decomposed into peptidoglycan, which can be degraded into a cell wall peptide with immunomodulatory activity and enter the cytosol, thereby activating NOD2.^93^ In general, the LRR domain of the NLR molecule folds to form a U-shaped configuration with the central NACHT domain, which inhibits its multimerization and makes the NLRs inactive.^94^ Once PAMPs directly or indirectly bind to the LRRs, the NLR molecule change their conformation, exposing the NACHT oligomerization domain, which triggers oligomerization, and the NLR molecule is activated.^95^ At the same time, the N-terminal effector domain is exposed, and through homotypic interactions, downstream adaptor molecules and signaling proteins with the same structure are recruited to initiate the corresponding signal transduction (Fig. 3).^96^ Although NOD1 and NOD2 do not have transmembrane domains, studies have shown that they are recruited into the plasma membrane and endosomal membrane, which is necessary for signal transduction.^97^ In this process, palmitoylation plays a vital role. The modification of NOD1/2 protein under the action of palmitoyltransferase ZDHHC5, which makes NOD1/2, possess the characteristics of rapid and reversible localization changes, which is necessary for membrane recruitment and inflammatory signal transduction.^98^ This study gives us a good enlightenment that the modification of PRRs may play a key role in the regulation of host innate immune signal.

### RIG-I-like receptors

**Fig. 4:**
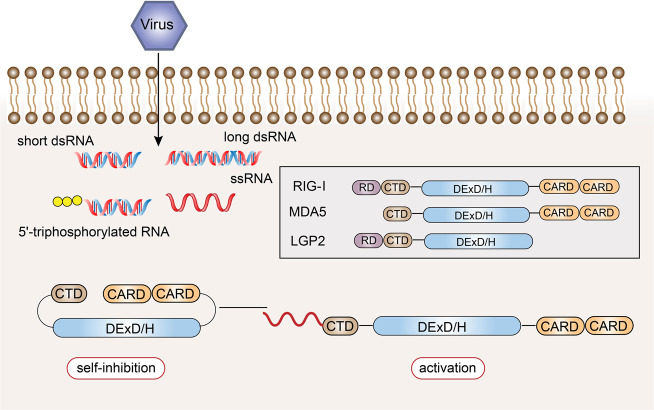
Structural features and ligand recognition mechanism of RLRs. The structure and functions of MDA5 are similar to those of RIG-I. However, MDA5 lacks the repressor domain, so it does not have self-inhibitory functions. LGP2 does not have CARD, and so it cannot transmit signals. The combination of viral RNA and CTD changes the conformation of RLRs

RLRs are also intracellular PRRs. In innate antiviral immunity, in addition to the recognition of viral nucleic acids by TLR7 and TLR9, most other types of cells recognize viral nucleic acids through RLRs to induce antiviral immune responses.^99,100^ The currently discovered RLR family members mainly include three: RIG-I, melanoma differentiation-associated gene 5 (MDA5), and laboratory of genetics and physiology 2 (LGP2) (Fig. 4).^101^

RIG-I was first discovered in acute promyelocytic leukemia cells induced by retinoic acid. In 2004, it was found that RIG-I could induce the expression of a reporter gene in the IFN-β promoter region, which confirmed its antiviral activity.^102^ The structure of the RIG-I protein consists of three parts.^103–105^ The middle part is the DexD/H helicase domain, which is the common domain of the RLR family, and has ATPase and helicase activities.^106–108^ The N-terminus of the RIG-I protein is composed of two caspase activation and recruitment domains in series,^109^ which are responsible for transmitting signals downstream.^110^ The C-terminus is composed of the repressor domain (RD) and the C-terminal domain (CTD), which can regulate its own state.^106,111^ The former can inhibit the activation of the receptor, and the latter is responsible for the recognition of viral RNA.^112,113^ In the resting state, CARD, CTD, and the helicase domain are folded, and RIG-I is in a self-inhibited state. During viral infection, the CTD of RIG-I recognizes viral RNA and undergoes a conformational change.^114^ RIG-I uses ATP hydrolase activity to expose and activate the CARD and multimerize, thereby recruiting downstream signaling linker molecules (Fig. 4).^115–117^

The structure and functions of MDA5 are similar to those of RIG-I, with the DexD/H helicase domain in the middle, two CARD at the N-terminus, and a CTD at the C-terminus; however, MDA5 lacks the RD, and so it does not have self-inhibitory functions. In contrast to other RLRs, LGP2 does not have CARD,^118,119^ and so it cannot recruit molecules of the same structure to transmit signals, but it can regulate the recognition of viral nucleic acids by RIG-I and MDA5, thereby preventing RLR-mediated resistance.^120–123^ LGP2 can negatively regulate RIG-I-mediated recognition of viral dsRNA, reduce the production of IFNs and inflammatory factors, and ultimately inhibit the antiviral innate immune response.^124^ LGP2 is also critical in the antiviral response mediated by MDA5.^125^ LGP2 exhibits a concentration-dependent conversion between MDA5-specific enhancement and interference.^126^ The latest research revealed a mechanistic basis for LGP2-mediated regulation of MDA5 antiviral innate immune responses. LGP2 facilitates MDA5 fiber assembly and is incorporated into the fibers, forming hetero-oligomers with MDA5.^127^ In addition, LGP2 can significantly induce the exposure of the CARD domain of MDA5.^128^ Under bacterial infection of the Indian major carp *Labeo rohita*, LGP2 gene expression was significantly increased after dsRNA and various PAMPs were stimulated, indicating that LGP2 can act as an antiviral and antibacterial cytoplasmic receptor.^129^

Although the RLR family members have similar structures, they recognize the RNA of different viruses through ligand-recognition domains.^130^ Both RIG-I and MDA5 can recognize viral dsRNA, but their recognition depends on the length of the dsRNA.^131^ RIG-I mainly recognizes viruses with relatively short dsRNA (<1000 bp), while MDA5 tends to recognize long-chain dsRNA (>1000 bp).^132^ Additionally, RIG-I mediates the antiviral response by recognizing the 5’-triphosphate RNA of viruses.^133^ The 5’-terminal triphosphate group can be recognized by RIG-I as a non-self component, but after posttranslational modification, this molecule cannot be recognized by RIG-I.^134^ Because host cell RNA needs to undergo different degrees of processing and modification after synthesis in the nucleus, these results indicate that RIG-I can distinguish viral dsRNA from endogenous RNA. In the cell, RIG-I mainly recognizes influenza virus,^135^ vesicular stomatitis virus,^136^ Sendai virus, and Japanese encephalitis virus,^137,138^ while MDA5 mainly recognizes small RNA viruses, such as poliovirus.^139,140^ MDA5 also participates in the synthesis of the dsRNA analog polycytidylic acid (poly I:C). Previous studies have shown that filamentous fibers are formed during the recognition of ligands by RIG-I and MDA5, and signaling pathways are initiated from the tail and inside of the viral dsRNA, respectively.^141^

Although it is mentioned in the “Toll-like receptors” section that TLR3, TLR7, TLR8, and TLR9 specifically recognize virus-derived nucleic acid molecules and bacterial nuclear components, they mainly appear in the endosomal membrane. RLRs can not only be expressed in cells infected by various viruses but also can directly recognize and perceive the virus products and virus particles that exist in the cytosol. Its antiviral significance cannot be ignored.^142^

### C-type lectin receptors

CLRs, which belong to phagocytic PRRs, are also a popular type of receptor under study.^143^ The function of phagocytic receptor is different from the receptor that activates cells by signal transduction. It recognizes and binds to PAMPs through PRRs and places pathogens in cytoplasmic vesicles for direct digestion and elimination to control infection.^144^ CLRs are a class of receptors that recognize carbohydrates on the surface of pathogenic microorganisms with the participation of Ca^+^.^145^ It is expressed on macrophages, dendritic cells (DCs), and certain tissue cells. The ability of CLRs to recognize carbohydrates existing on self and non-self structures is mediated by carbohydrate recognition domain (CRD).^146^ The CRD of CLRs is a compact spherical structure, and this region is called C-type lectin-like domain (CTLD).^147,148^ Depending on the location of the protein on the cell membrane, CLRs are divided into transmembrane receptors and secretory receptors.^146,149,150^ The main representative of secretory receptors is collagen lectin family (under the “Extracellular pattern recognition molecules”).^151^ Transmembrane receptors can be divided into type I and type II according to their topological structure.^152,153^ The N-terminal of type I receptors points to extracellular and contains multiple CRDs, while the N-terminal of type II receptors points to intracellular and contains only one CRD.^154,155^ It has been shown that the vast majority of CLRs are involved in the presentation of antigens as active membrane-associated receptors, and CLRs are mainly expressed on antigen-presenting cells such as DCs and macrophages.^145^ CLRs are circular structures connected by two disulfide bonds.^156^ CLRs contain at least one CTLD outside the cell, while the intracellular domain is different.

Mannose receptors (MRs) belong to membrane CLRs, which are single-chain transmembrane molecules.^157–159^ The extracellular segment of MR consists of two parts: one is the proximal membrane end with eight consecutive CTLDs, which is responsible for the endocytosis and transport of the ligand; the other is the distal membrane end of the cysteine-rich lectin domain, which recognizes sulfation of carbohydrate conjugates.^160^ The endogenous ligands of MR are lysosomal hydrolase and myeloperoxidase, as well as the mannan-rich structure expressed by pathogens.^161,162^

Dendritic cell-associated C-type lectin (Dectin)-1 and Dectin-2 are typical representatives of the CLR family.^163,164^ Dectin-1 is a type II transmembrane protein expressed in DCs, macrophages, neutrophils, and monocytes.^165^ The extracellular region is a CTLD. The intracellular tail is connected to an immunoreceptor tyrosine-based activation motif (ITAM),^166^ indicating that the receptor also has a signal transduction function. Dectin-1 can identify a variety of fungi,^167^ including yeast,^168^ *Candida albicans*,^169,170^ *Pneumocystis carinii*,^171,172^ Cryptococcus,^173,174^ and Aspergillus.^175,176^ The ligand of Dectin-1 is β-1,3-glucan, which can activate downstream signals through tyrosine kinase-dependent and tyrosine kinase-independent pathways after recognition and binding of the ligand.^177,178^ Glycosylation is an important modification of the posttranslational modification of proteins (including antibodies),^179^ which can significantly change the structure and function of proteins or antibodies, so it is also a key mechanism for the immune system to regulate biological activity.^180^ Abnormal glycosylation is usually associated with malignant tumors.^179^ Therefore, the identification of molecules that bind glycosylated glycans can provide a new way for the treatment of human infectious and malignant diseases. Studies have found that Dectin-1 can recognize aromatic amino acids adjacent to the N-terminal asparagine at the glycosylation site as well as the core fucose on IgG antibodies, which do not compete for the same protein binding site for β-glucan, so Dectin-1 can regulate the immune response induced by IgG by combining with core fucose.^181^

Dectin-2, which is different from Dectin-1, does not contain the ITAM sequence and has no signal transduction function.^182^ Dectin-2 mainly recognizes α-mannan in the fungal cell wall and recognizes the *Schistosoma mansoni* egg antigen.^183,184^ The molecular mechanism by which Dectin-2 recognizes the binding ligand has always been the focus of research. Decout et al.^185^ found that the stimulation of Dectin-2 by purified *Mycobacterium tuberculosis* mannose-capped lipoarabinomannan requires the (α1 → 2)-linked mannosides forming the cap. Besides, Dectin-2 can also recognize lipoglycans from other bacterial species.^185,186^ From the perspective of the relationship between the structure and function of the above two ligands, dimannoside caps and multivalent interaction are necessary for Dectin-2 to recognize binding ligands and conduct signals.^187^

### AIM2-like receptors

ALRs are a new type of PRRs that can recognize intracellular DNA.^188,189^ The C-terminus is the DNA-binding domain HIN-200, and the N-terminus is the PYD.^189–192^ The HIN-200 domain recognizes double-stranded DNA and binds to it. The N-terminal PYD binds to the PYD of apoptosis-associated speck-like protein containing CARD (ASC),^193,194^ thereby promoting the formation of inflammasomes and the maturation and release of IL-1β and IL-18.^195^ Both the DNA-binding affinity of AIM2 and the activity of its inflammasome depend on dsDNA, and it can assemble into filamentous structures along dsDNA. However, without dsDNA, it can also form filaments at high protein concentrations.^196–198^ ALRs can not only participate in the innate immune response but also regulate apoptosis, which is related to the occurrence and development of tumors.^199^

### Extracellular soluble pattern recognition molecules

The initiation of the innate immune response depends on the recognition of PAMPs by pattern recognition molecules (PRMs), including cell PRRs and extracellular soluble PRMs. They are a class of free receptors that can play an antibacterial effect in serum.^200^ Although the pattern recognition of innate immunity does not have the antigen specificity of the adaptive immune response, some PRMs produced by the body after infection by pathogenic microorganisms will exist in the serum. Once the new pathogens invade, they can also bind to the pathogen like an antibacterial molecule and play an effective function. Unlike cell-related PRRs, extracellular soluble PRMs are an important part of non-specific humoral immunity.^201^ Extracellular soluble PRMs are composed of different molecular families, mainly including pentraxin,^202^ collectin, and ficolin.^203^ They generally function in two ways: one is that they recognize various pathogenic factors and eliminate them through complement activation,^204,205^ opsonization,^206^ aggregation, and neutralization of inflammatory regulation; the other is that they interact with cell-related PRRs and regulate their functions to jointly regulate innate immune response.^207^

Pentraxin is characterized by the aggregation of five molecules and is highly conserved in evolution, including two families of short molecules and long molecules.^208–211^ The family of short molecules is called acute phase proteins, which is represented by C-reactive protein (CRP)^212–214^ and serum amyloid P component^215,216^ in humans and mice, respectively. These molecules are mainly produced by the liver under the stimulation of inflammatory signals and interleukins. They are non-specific proteins that reflect the systemic inflammatory response. Serum levels increase rapidly after the body is infected or injured. CRP generally binds to phosphocholine expressed on the surface of pathogenic microorganisms in a Ca^+^-dependent manner.^217^ SAA can bind to the outer membrane protein A of bacteria and interact with TLRs.^218,219^ In clinic, SAA and CRP are usually used as auxiliary diagnostic indicators for infectious diseases, but studies have shown that they also have diagnostic value in non-infectious diseases and can be used as disease classification markers.^220,221^ The representative of the pentraxin long molecule family is PTX3,^222^ which is unique in that it has a long N-terminal domain. PTX3 is produced by dendritic cells, monocyte macrophages, epithelial cells, smooth muscle cells (SMCs), and endothelial cells under the regulation of a variety of inflammatory factors.^223^ PTX3 is involved in the defense of selected pathogens and the regulation of inflammation.^224–226^ Due to its expression increases sharply under the conditions of inflammatory stimulation, PTX3 can become a biomarker of general acute inflammation and a variety of tumors.^227^ In coronavirus disease 2019 (COVID-19) patients, circulating and lung bone marrow monocytes and endothelial cells express high levels of PTX3, and PTX3 plasma concentration can serve as an independent strong prognostic indicator of short-term mortality in COVID-19.^228,229^

Collectin mainly includes mannose-binding lectin (MBL) and surfactant protein (SP).^151,230^ MBL is formed by connecting multiple homotrimers. Each component of the trimer includes a CRD, an alpha helix, and a main stem formed by spirals of collagen.^231,232^ The main stem of collagen gathers each trimer into bundles. MBL is composed of six CRDs.^151^ The end of CRD can identify the sugar structure on the surface of various pathogens, such as mannose, fucose, glucose, etc.^233–235^ The pathogens involved include yeast, parasites, Gram bacteria, and so on.^236–240^ When the distance of each CRD between the same trimer or adjacent trimers is 45 Å, it is most conducive to ligand binding.^241^ The other family members include A and D,^242^ which exist on the surface of the alveoli and are important innate immune defense molecules in the lungs. Both of them are composed of N-terminal region, CRD, neck region, collagen-like region, and other parts.^243^ CRD recognizes and binds glycosyl groups. The biological significance is that they can selectively identify microbial carbohydrate structures that are harmful to themselves.^244,245^

The domain of ficolin is similar to collectin, but it recognizes a variety of bacteria with a fibrinogen-type carbohydrate recognition structure.^246,247^ Its ligands are *N*-acetylglucosamine and LTA, a cell wall component of Gram-positive bacteria.^248,249^

## Signaling pathways of PRRs

There are three main types of molecules involved in signal transduction: protein kinases, adaptor proteins, and transcription factors. Although PRRs are activated by their respective ligands in different subcellular structures with different mechanisms, the three main types of molecules involved in signal transduction have similar structures and functions, and the signals they transmit are cross-talking, which can converge into several common signaling pathways.

### The NF-κB signaling

**Fig. 5:**
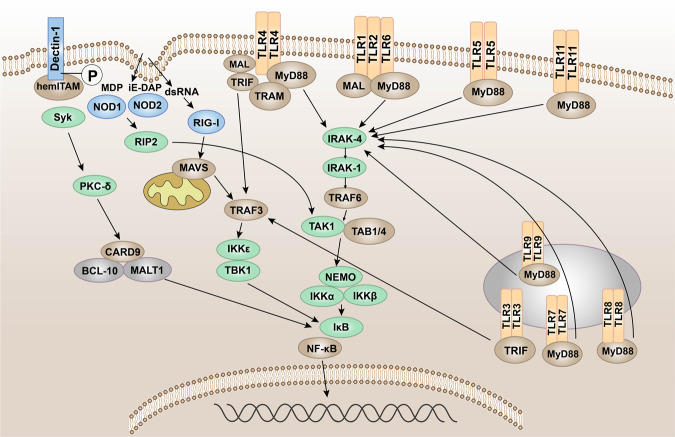
Pattern recognition receptor-mediated NF-κB signaling. The NF-κB protein can regulate gene expression and affect various biological processes, including innate and adaptive immunity, inflammation, stress response, B cell development, and lymphoid organ formation. TLRs, NLRs, RLRs, and CLRs can generally phosphorylate IκB protein, which inhibits the activation of NF-κB protein, thereby promoting the transcription and activation of inflammatory genes

The transcription factor NF-κB is named after it was first discovered to be involved in the transcription of B cell κ chain genes.^250^ NF-κB is a heterodimer composed of two molecules, p50 and p65, and is inactive due to binding to the inhibitory protein IκB under normal conditions. NF-κB plays a key role in the process of cellular inflammation and immune response,^251,252^ and its mediated signal pathways are commonly seen in the activation of various immune cells, including signal transduction initiated by PRRs in innate immunity (Fig. 5).^253^

In the signal transduction initiated by TLRs,^45^ after TLRs recognize and bind the corresponding PAMPs and DAMPs, the TIR domains conduct signals by binding to different receptor adaptor proteins in the cytoplasmic region.^254,255^ Depending on the different adaptor proteins, TLR signaling can be divided into MyD88-dependent and MyD88-independent pathways.^256^ MyD88 has a TIR domain at the C-terminus and a death domain at the N-terminus and is the linker molecule in most TLR signal transduction pathways.^257^ The current research indicated that, in the MyD88-dependent pathway, MyD88 signaling mainly leads to the production of pro-inflammatory cytokines, such as tumor necrosis factor (TNF), IL-6, IL-1, and chemokines.^258–260^ The C-terminus of MyD88 binds to the intracellular TIR domain of TLRs, and the N-terminus of MyD88 recruits IL-1R-related kinase 4 (IRAK4)^261^ and activates IRAK1 and IRAK2 through autophosphorylation of its central kinase domain. Then ubiquitin ligase TNF receptor-associated factor 6 (TRAF6) is recruited to form a complex with transforming growth factor (TGF)-β-activated kinase 1 (TAK1) and two TAK-binding proteins (TAB1 and TAB4). TRAF6 is degraded due to its own ubiquitination.^262,263^ The TAK1–TAB1–TAB4 complex activates the IκB kinase (IKK) complex through phosphorylation. The latter phosphorylates IκB and degrades itself by ubiquitination. NF-κB is released and translocated to the nucleus, thereby regulating the transcription of inflammatory genes.^264,265^

In the signal pathway mediated by NLRs, when the bacterial component invades the cell, NOD1 and NOD2 recognize the bacterial iE-DAP and MDP, respectively.^266,267^ And then NOD-like receptors are activated, self-dimerize, and recruit downstream receptor-interacting serine–threonine protein 2 (RIP2) through its CARD.^268^ Activated RIP2 gathers downstream TAK1, TAK1-binding protein 1, and the NF-κB essential modulator/IKKα/IKKβ complex, and the former activates IKKα/IKKβ,^269^ thereby activating the transcription of NF-κB and promoting the release of pro-inflammatory factors.

When virus invades cells, RIG-I and MDA5 recognize the corresponding viral RNA through the CTD and undergo conformation changes.^270^ Activated RIG-I and MDA-5 induce downstream signal transduction by binding with mitochondrial antiviral signaling protein (MAVS). MAVS is an important adaptor protein for downstream signal transduction. The N-terminus contains a CARD-like domain, which binds to RIG-I and MDA-5 through the CARD–CARD interaction.^271,272^ The proline-enriched domain in MAVS can interact with a series of downstream signaling molecules, such as TRAF3 and 6,^273^ and activate the protein kinase IKK, which causes phosphorylation of IκB,^265^ and then IκB is ubiquitinated and degraded by proteases, activating the NF-κB pathway.^274^

Different from other typical PRR-mediated signaling pathways, spleen tyrosine kinase (Syk) can be activated by associating with the phosphorylated ITAM motif of CLRs.^275^ In the Dectin-1/Syk pathway, Syk activates protein kinase C-δ, which mediates the phosphorylation of CARD9.^276^ This allows CARD9 to bind to B cell lymphoma 10^277^ and para-aspase mucosa-associated lymphoid tissue lymphoma translocation protein 1, forming a three molecular structure that can typically activate NF-κB.^278^

### The mitogen-activated protein kinase (MAPK) signaling

MAPK is a group of serine–threonine protein kinases that can be activated by different extracellular stimuli,^279^ such as cytokines, neurotransmitters, hormones, cell stress, and cell adhesion. The MAPK pathway is one of the common intersections of signal transduction pathways, such as cell proliferation, stress, inflammation, differentiation, functional synchronization, transformation, and apoptosis.^280,281^ It is an important transmitter of signals from the cell surface to the inside of the nucleus.

In the MyD88-dependent pathway of TLRs, IRAK-1 is activated by phosphorylation and interacts with TRAF6. In addition to activating the IKK complex, it can also cause the activation of MAPKs (c-Jun N-terminal kinase (JNK), p38 MAPK).^282^ In addition, when bacterial components invade cells, NLRs are activated, recruiting downstream CARD9, thereby activating p38, JNK, and finally activating the MAPK pathway^283^ to promote the release of pro-inflammatory factors.

### The TBK1–IRF-3 signaling

**Fig. 6:**
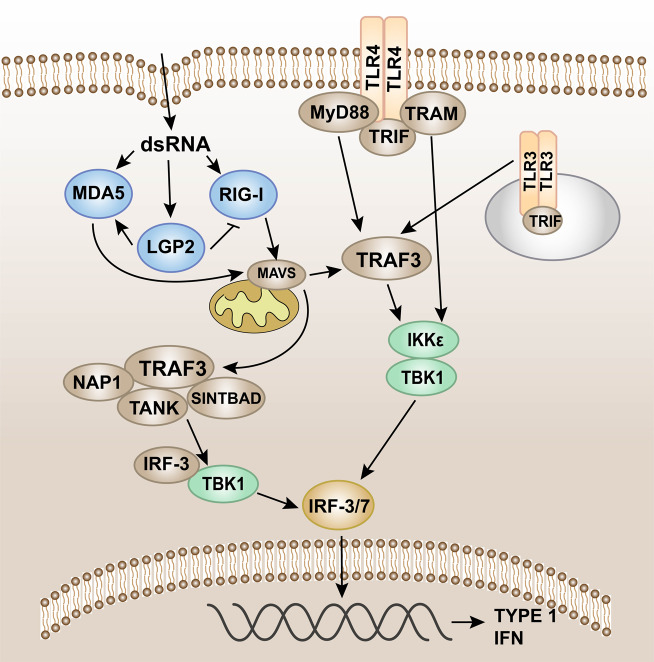
Pattern recognition receptor-mediated TBK1-IRF-3 signaling. Intracellular induction of pathogens is carried out through the detection of foreign molecular components (including cytoplasmic viral and bacterial nucleic acids). Once detected, the innate immune system induces type I interferon (IFN) production through the TANK-binding kinase 1 (TBK1)-interferon regulatory factor-3/7 (IRF-3/7) pathway. IRF-3/7 can be activated through two innate immune antiviral signal pathways, TLR3/TLR4-TIR domain-containing adaptor protein-inducing interferon β (TRIF) and RIG-I-MAVS, and then dimerize and merge into the nucleus to work

IRF-3 is a key transcription factor that promotes the synthesis of type I IFN and plays an important role in the antiviral innate immune response.^284^ IRF-3 can be activated through two innate immune antiviral signal pathways, TLR3/TLR4-TIR domain-containing adaptor protein-inducing interferon β (TRIF) and RIG-I-MAVS,^285^ and then dimerize and merge into the nucleus to work (Fig. 6).^286^

The adaptor protein in the MyD88-independent pathway is TRIF. The TRIF axis mainly induces the expression of type I IFNs.^287^ After the receptor is recognized and combined with the ligand, the pathway is activated by TRIF and TRAF3, leading to the recruitment of IKKε/TANK-binding kinase 1 (TBK1),^288^ phosphorylation of IRF3, and the activation of type I IFN genes, which promotes the expression of IFN-α and IFN-β, and exerts antiviral effects (Fig. 6).^289–291^

RLRs such as RIG-I and MDA5 can detect viral nucleic acid. MDA5 and RIG-I will interact with the shared caspase recruitment domain to induce MAVS to dimerize and bind to TRAF3.^134,140,292^ In turn, TRAF3 recruits the adaptor proteins TANK, NAP1, and SINTBAD. TANK connects upstream RLR signal transduction to TBK1, which induces phosphorylation of IRF-3. IRF-3 phosphorylation and subsequent dimerization induce IRF-3 nuclear translocation, leading to type I IFN gene expression (Fig. 6).^192,293,294^

### The inflammasome signaling

**Fig. 7:**
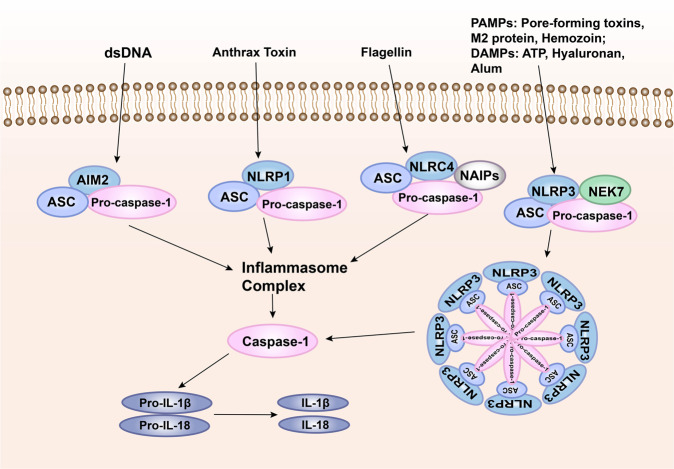
Pattern recognition receptor-mediated inflammasome signaling. One way for pathogenic microorganisms to induce inflammation is by activating inflammasomes, which are multi-protein complexes assembled by PRRs in the cytoplasm and activate caspase-1 and subsequent activation of pro-inflammatory cytokines IL-1β and IL-18. The inflammasome complex usually contains cytoplasmic PRRs, adaptor protein (ASC), and pro-caspase-1. Many different inflammasome complexes have been detected, each with unique PRRs and activation triggers

Inflammasome is the multi-protein complex assembled by PRRs in the cytoplasm and is an important part of the innate immune system.^295^ The inflammasome can recognize PAMPs or DAMPs and recruit and activate Caspase-1. The activated Caspase-1 spliced proIL-1β/proIL-18 into the corresponding mature cytokine.^193,296^ There are five main types of inflammasomes that have been discovered, namely, NLRP1 inflammasome,^297^ NLRP3 inflammasome,^298^ NLRC4 inflammasome,^299,300^ IPAF inflammasome, and AIM2 inflammasome.^301^ Known inflammasomes generally contain ASC, caspase protease, and a protein of the NLR family (e.g., NLRP3) or HIN-200 family protein (e.g., AIM2). Taking NLRP3 as an example,^302^ the dimerization of NLRP3 under the action of intracellular PAMPs or DAMPs makes the two PYDs to polymerize. With the help of homotype interaction, NLRP3 binds and activates the ASC complex with both PYD and CARD domains, which reactivates the effector complex composed of CARD and caspase-1. In this way, NLRP3 (LRR + NACHT + PYD), ASC (PYD + CARD), and the effector complex (CARD + Caspase-1) together constitute the inflammasome, which produces important pro-inflammatory factors.^303–305^ After AIM2 recognizes cytoplasmic dsDNA, it also uses inflammasomes to produce IL-1β and IL-18. After AIM2 recognizes cytoplasmic dsDNA, it also produces IL-1β and IL-18 through the inflammasome pathway (Fig. 7).^306^

Innate immunity not only plays a role in controlling the infection and spread of pathogens in the early stage of infection but also plays an important role in initiating and regulating adaptive immunity.^12,307^ Innate immune cells produce different types of cytokines through signal transduction initiated after PRRs recognize PAMPs, which directly affect the differentiation of T helper type 1 (Th1), Th2, Th17, and other subgroups in adaptive immunity.^15^ For example, pathogenic microorganisms activate macrophages to secrete IL-6, TGF-β, IL-23, and other cytokines, which promote Th17 response, leading to excessive immune-inflammatory effects and tissue damage, or activate NK cells to secrete IFN-γ, and then activate macrophages to secrete IL-12, promote the differentiation of Th0 into Th1, promote cellular immune response, and effectively eliminate viral infections.^13,308^ Therefore, the immune system is a system of mutual influence. Any anti-infection process is completed by mutually activating or inhibiting of different components. These components are as small as each cytokine and as large as the immune system.

## PRR-related diseases

### PRRs and cancers

The inflammatory microenvironment of tumor constitutes the barrier for tumor growth, which is conducive to tumor formation and development.^309^ PRRs are widely expressed in a variety of tumor tissues, such as colon cancer, lung cancer, breast cancer, gastric cancer, melanoma, and so on.^310,311^ The activation of PRRs on the surface of tumor cells can induce the expression of a large number of cytokines, chemokines, hormones, and vascular-promoting factors, which is one of the important factors to induce the formation of tumor inflammatory microenvironment and promote the development of tumor.^312,313^ At the same time, the activation of PRRs on immune cells can induce antigen-presenting cells including DCs, tumor-associated macrophages, and B cells to activate tumor-specific T cell responses or enhance the antitumor effects of phagocytes. These also indicate that the role of PRRs in immunotherapy against tumors is very important and might represent a new strategy for patients with tumors.^314,315^

We can see from the data in GEPIA^316^ (http://gepia.cancer-pku.cn/) that TLR level is significantly increased in tumors, including glioblastoma multiforme, brain lower-grade glioma, kidney renal clear cell carcinoma, acute myeloid leukemia (AML), and pancreatic adenocarcinoma (PAAD). NOD1/2 are highly expressed in AML and PAAD, while RLRs are highly expressed in AML, PAAD, diffuse large B cell lymphoma, head and neck squamous cell carcinoma, and thymoma.

#### Colorectal cancer (CRC)

CRC, including colon cancer and rectal cancer, is one of the most common gastrointestinal malignancies in clinical practice, and it is also one of the cancers that seriously endanger human health.^317,318^ Intestinal mucosal epithelial cells and immune cells recognize intestinal microorganisms and their products through TLRs.^319–322^ TLR2 can recognize peptidoglycans and lipopeptides that infect intestinal epithelial bacteria and produce anti-infection and other immune-protective effects.^323^ Studies have shown that the expression level of TLR2 protein in colon cancer is significantly upregulated compared with normal epithelial tissues,^324^ and the use of TLR2 agonists significantly enhances the proliferation, migration, and invasion capabilities of colon cancer cells.^325^ TLR4 is highly expressed on the surface of colon cancer cells. After stimulation and activation, it can induce a variety of immunosuppressive factors, thus promoting the proliferation and immune escape of colon cancer cells.^326^ First, TLR4 can produce trophic factors and vascular growth factors through the TLR4/MyD88/NF-κB signaling pathway, thereby promoting tumor cell invasion.^327,328^ Second, TLR4 can promote tumor proliferation through TLR4/Cyclooxygenase 2 (COX2)/prostaglandin E2 (PGE2). PGE2 is an important cell growth and regulatory factor. After binding to specific receptors, it plays a key role in mediating a series of cell activities, such as cell proliferation, differentiation, and apoptosis, and has immunosuppressive and anti-inflammatory effects. COX2 is the rate-limiting enzyme of prostaglandin synthesis, and it is also highly expressed in inflammation, tumor, and other pathological states.^329^ Hsu et al.^330^ found that knocking out the mouse TLR4 gene significantly reduced the expression of COX2 and PGE2 in the intestinal mucosa; after administration of PGE2, the expression of COX2 in the intestinal mucosa increased significantly and promoted the occurrence of intestinal tumors. After administration of PGE2, the expression of COX2 in the intestinal mucosa increased significantly and promoted the occurrence of intestinal tumors. At the same time, it has also been found to promote the expression of amphiregulin and epidermal growth factor receptor (EGFR) in the intestinal mucosa. Finally, the study showed that the abnormal expression of TLR4 in CRC caused by chronic inflammation of the intestine can significantly enhance the expression of PGE2, the upregulation of COX2, and the phosphorylation of EGFR in intestinal mucosal cells, thereby positive feedback promotes the proliferation of tumor cells. TLR5 also plays an important role in tumor immunotherapy.^331^ In mouse xenograft models of human colon cancer, flagellin around the tumor activates TLR5 to inhibit tumor growth and promote tumor apoptosis.^332^ In addition, TLR9 is expressed on the surface of the mesentery, which maintains intestinal homeostasis and repairs intestinal damage by generating an immune response.^333^ TLR9 relies on the MyD88 pathway to induce downstream signals to recruit many inflammatory factors, such as IL-8, TGF-β, PGE2, and other immunosuppressive molecules,^334^ leading to the continuous development of inflammation, resulting in immune escape, and promoting the unlimited proliferation of tumor cells. After TLR9 recognizes the exogenous ligand, it upregulates the expression of NF-κB signaling factor. Once this pathway is opened, it may induce the secretion of matrix metalloproteinase-13 (MMP-13) and the activation of intercellular adhesion molecule-1, thus promoting the metastasis of tumor cells.^335,336^ At the same time, the metastatic tumor cells are better adapted and combined with the cell matrix at the metastasis, and the stability of tumor cell metastasis is enhanced. Although TLRs have been shown to enhance colon cancer metastasis, inhibiting these receptors cannot completely hinder tumor progression. Surprisingly, NOD1 is highly expressed in human CRC and its cell lines. After being activated by C12-iE-DAP, it mainly enhances the adhesion, migration, and metastasis of CRC cells through the p38 MAPK pathway.^337^

#### Hepatocellular carcinoma (HCC)

HCC is the most common type of primary liver cancer. Among its many influencing factors, inflammation is one of the main reasons that induce liver cancer.^338^ The expression of TLR2 in liver cancer tissues is significantly higher than that in normal liver tissues, and the expression of TLR2 protein is related to some mutant genes that lead to the occurrence of HCC, such as p53, PIK3CA, and β-catenin.^339–342^ In addition, Chew et al. revealed that the expression of TLR3 has independent effects on tumor parenchyma and infiltrating NK cells, and the expression of these two parts is related to inhibiting tumor cell proliferation, promoting tumor cell death, and prolonging the survival rate of patients.^343^ This indicates that TLR3 may directly act on tumor parenchymal cells, promote the recruitment and activation of NK cells, and exert antitumor effects. More and more evidences show that LPS plays a role in the development of HCC. Zhou et al.^344^ found that LPS activates the TLR4–AKT–SOX2 signaling pathway of liver cancer cell lines to improve the ability of cancer stem cells; Lin et al.^345^ found that there is a positive feedback loop of COX-2/PGE2/signal transducer and activator of transcription factor 3 (STAT3) activated by LPS in liver cancer cells, which regulates the expression of genes related to tumor proliferation, differentiation, and apoptosis. In the latest research on the treatment of liver cancer, it is found that the antitumor effect of TLR9 agonist combined with anti-PD-1 antibody or anti-PD-L1 is significantly better than single-agent therapy.^346^ The activation of TLR9 inherent in liver cancer cells regulates the autoarylation and ubiquitination of poly(ADP-ribose) polymerase-1 and the phosphorylation of STAT3, which together upregulate the expression of PD-L1 and eventually induce immune escape. Although TLRs have been reported to be associated with chronic inflammation of the liver, whether they promote the development of HCC remains uncertain. Song et al.^347^ found that the deficiency of TLR4, TLR9, and their downstream molecule MyD88 in a mouse model characterized by hepatic deletion of TAK1 could block the liver inflammation–fibrosis–cancer axis and reduce liver injury and tumor growth. For TLR3, the downregulation of TLR3 in HCC patients leads to poor prognosis (e.g., defective immune cell recruitment and lack of killing of transformed hepatocytes), leading to protection of transformed hepatocytes from apoptosis, thereby promoting the occurrence of liver cancer.^348^ Therefore, the expression of TLR3 may become a useful clinical treatment monitoring marker.

In addition to the above reasons, there is now more and more evidence that the imbalance of the gut–hepatic axis may also play a role in the occurrence of HCC.^349^ Zhou et al.^350^ discovered that NOD2 acts as a bacterial sensor, linking gut-derived microorganisms to the occurrence of HCC through a known mechanism and a newly discovered mechanism. The known mechanism is that NOD2 activates NF-κB, JAK2/STAT3, and MAPK pathways in a RIP2-dependent manner, leading to liver inflammation.^351^ It is worth noting that activated NOD2 can also act as the initiator of the nuclear autophagy pathway that does not depend on RIP2, thereby promoting the degradation of the nuclear component lamin A/C, leading to damage to DNA damage repair mechanisms and increased genomic instability, which eventually leads to the occurrence of HCC.^350^ Meanwhile, the study found that the expression of ALRs was negatively correlated with tumor volume, stage, and metastasis of HCC patients. The researchers proposed that overexpression of ALRs in HCC cells could increase the expression of caspase-1 and IL-1a, and the release of lactate dehydrogenase was also observed,^352^ which was a marker of the initiation of apoptosis. Thus, ALRs may play an antitumor role by promoting tumor cell apoptosis.

#### Breast cancer

Breast cancer is one of the most common malignant tumors in the female population. It has a strong ability to invade and metastasize.^353^ It can metastasize to the liver, lung, brain, bone, and other organs, forming complications and increasing the difficulty of treatment. Studies have shown that the promotion of the TLR2 signaling pathway on the metastasis and invasion ability of human breast cancer cells is achieved by upregulating the secretion of inflammatory cytokines.^354^ LTA, a TLR2 specific ligand, can significantly promote the secretion of tumor metastasis-related factors IL-6, TGF-β, and vascular endothelial growth factor (VEGF) in breast cancer cells,^355^ thereby promoting the proliferation and metastatic invasion of breast cancer cells, and this promotion is related to the level of TLR2 expression.^356,357^ In addition, the activation of TLR4 can increase the secretion of IL-6 and IL-10 of cancer cells and induce the production of more MMP-2, MMP-9, and VEGF,^358,359^ which can significantly enhance the invasion ability of breast cancer. It has been reported that activation of TLR4 on metastatic breast cancer cells can regulate the expression of integrin, which can promote its adhesion and invasion.^360^

#### Head and neck squamous cell carcinoma

Squamous cell carcinoma, also known as epidermal carcinoma, is a malignant tumor occurring in the epidermis or adnexal cells.^361,362^ It is more common in the parts covered by squamous epithelium, such as skin, mouth, lip, esophagus, cervix, vagina, etc.^363,364^ TLR2, TLR4, and TLR9 are expressed in primary tumors, neck metastases, and recurrent tumors of oral tongue squamous cell carcinoma (OTSCC), and their expression varies from the tumor surface to the invasive front, which may be one of the important factors to promote the invasion of OTSCC.^365,366^ NOD1 and NOD2 genes are expressed in the human oral squamous cell carcinoma (OSCC) cell line YD-10B, and they may trigger immune responses through the MAPK pathway. Surprisingly, the study revealed that stimulation by the NOD2 agonist MDP can inhibit cell growth by inducing apoptosis. These findings provide the potential value of MDP as a new candidate for OSCC antitumor drugs.^367^

### Respiratory diseases

*Aspergillus fumigatus* is a fungus widely distributed in nature. It can easily invade the respiratory tract and cause bronchitis and pneumonia in patients.^368,369^ For the allergic lung inflammation caused by Aspergillus, the recognition of PAMPs by the body’s dendritic cells is mainly negatively regulated through the TLR2-MyD88 pathway. The results showed that PAMPs recognized by TLR2 upregulated IL-10 and decreased the recruitment of pulmonary eosinophils, thus downregulating Th2 response.^370,371^ In allergic asthma, TLR9–IL-2 affects the Th2 response by regulating the expression of IL-17A, so small molecule inhibitors targeting TLR9 may become a new treatment strategy.^372^

### Nervous diseases

The connection between innate immunity and nervous system is becoming more and more complex and close.^373–375^ Studies have shown that NOD1/NOD2 may be a new target for the treatment of stress-related gut–brain diseases.^376^ The gut–brain axis is a biochemical signal of the digestive tract and central nervous system, which affects all events from brain development to the progression of neurological diseases. The hypothalamic–pituitary–adrenal axis (HPA) is one of the main pathways of gut–brain axis signal transmission.^377^ It has been reported that the immune system plays a key role in brain function and stress response. NLRs are PRRs expressed in the gut and brain. The lack of NOD1 and NOD2 affects the serotonergic signaling of gut and brain, the proliferation of hippocampal cells, and the maturation of neurons, which makes the mice lacking both of them vulnerable to HPA overactivation under stimulation, thus showing anxiety, cognitive impairment, and depression.^378–380^ TLR4 is very important in the process of neuropathic pain caused by infection and sterile neuronal injury. TLR4 exerts its effects through the activation and nuclear localization of NF-κB and the production of pro-inflammatory cytokines, which can activate pain receptors to cause neuropathic pain.^381,382^ Relevant studies have revealed for the first time that lysozyme acts as an endogenous ligand for activating TLR4 in sterile nerve injury, thereby promoting neuronal excitement and neuropathic pain.^383^ The identification of lysozyme as DAMPs has improved our understanding of neuroinflammation and opened up prospects for the treatment of neuropathic pain.

### Digestive diseases

Newborns with chronic obstructive jaundice make their livers prone to cholestatic liver disease, and biliary atresia (BA) accounts for half of the cases.^384^ Viruses have always been considered as the causative pathogen of this disease, and the role of TLRs in the pathogenesis and progression of BA has been determined.^385^ Subsequent studies have shown that activation of TLR7 can induce type 1 IFN signal transduction, apoptosis, and dysplasia of the neonatal liver and biliary system. This new discovery reveals the pathogenesis of neonatal cholestatic liver disease.^386^

The expression of TLR5 is closely related to various infectious diseases caused by bacteria.^387,388^ The lack of TLR5 can cause changes in the intestinal flora and cause colitis.^389^ The protein–protein interaction between TLR5 and flagellin plays an important role in pathogen defense, immune diseases, and tumors. 4-((4-benzyl-5-(pyridin4yl)-4H-1,2,4-triazol-3-yl)thio)pyrido[3’,2’:4,5]thieno[3,2-d] Pyrimidine (TH1020) is a small molecule inhibitor identified through high-throughput screening, which can disrupt the association between TLR5 and flagellin, and provides a lead compound for new therapies against TLR5.^390,391^

Alcoholic liver disease is a liver disease caused by long-term heavy drinking. The initial stage usually manifests as fatty liver, which can then develop into alcoholic hepatitis, liver fibrosis, and cirrhosis.^392,393^ Patients with advanced alcoholic cirrhosis are more susceptible to infection.^394^ This phenomenon is related to multiple organ failure and immunodeficiency and is usually manifested as insufficient antibacterial activity of neutrophils.^395^ The neutrophil function to resist microbial infections needs to generate reactive oxygen species through NADPH oxidase 2. Rolas et al. found that, in patients with alcoholic liver cirrhosis, the lack of catalytic core flavocytochrome b558 (gp91phox, p22phox) and p47phox of the NADPH enzyme may be a new factor that patients are susceptible to infection. What is surprising is that the activation of TLR7/8 can reverse the expression and activity of the deficient gp91phox, which provides a direction for restoring the antibacterial response of immunodeficiency patients.^396,397^

### Cardiovascular diseases

Atherosclerosis is a chronic inflammatory disease caused by plaques composed of lipids, cholesterol, calcium, and other substances in blood vessels.^398,399^ It has been reported that TLRs are extensively and deeply involved in the process of atherosclerosis.^400,401^ TLR7 has been identified as a good prognosis marker for patients with severe atherosclerosis. TLR7 in the plaque produced in atherosclerotic lesions will secrete IL-10 and TNF-α after being stimulated by ligand. Studies have found that TLR7 may regulate inflammation in atherosclerosis by inhibiting the effects of pro-inflammatory cytokines.^402^ In addition to the production of plaque, cell-free DNA (cfDNA) is also released in atherosclerotic lesions. TLR9 recognizes cfDNA and plays a key role in the development of vascular inflammation and atherosclerosis by promoting the pro-inflammatory activation of macrophages.^403^ The formation of initial atherosclerotic plaques is caused by the interaction of macrophages and endothelial cells, macrophage infiltration, and other factors that lead to an increase in neointima.^404,405^ It is worth noting that studies have shown that TLR5-mediated activation of NADPH oxidase 4 (Nox4) can regulate the migration of SMCs and promote the expression of pro-inflammatory cytokines, which may contribute to the formation of atherosclerotic plaques. This indicates that the flagellin–TLR5–Nox4 cascade is of great significance in atherosclerotic intimal hyperplasia.^406,407^ In addition, TLR2 activates p38 and extracellular signal-regulated kinase 1/2 signals, thereby upregulating IL-6-mediated receptor activator of NF-κB ligand and downregulating osteoprotegerin. These will make the cartilage formation of vascular SMC transdifferentiate, leading to vascular calcification.^408^ The expression of TLR3 is reduced in the lung tissue and endothelial cells of patients with pulmonary hypertension, and its deficiency increases the susceptibility to apoptosis and pulmonary hypertension.^409^ In addition to TLRs, the lack of NOD1 and NOD2 can lead to lipid deposition of atherosclerotic plaques and the reduction of inflammatory cell infiltration, so it has been identified as pre-disease factors.^410^

Human brain pericytes (HBPs) are an important component of the microvascular wall and contribute to the integrity of the blood–brain barrier (BBB).^411^ It has been found that TLR4 and NOD1 are expressed in HBP, which also reveals that HBP has the ability to sense systemic infection or blood-borne PAMPs. HBP can perceive Gram-negative bacteria to protect BBB through different pathways mediated by TLR4 and NOD1, or it can trigger paracrine signaling pathways by releasing chemokines and cytokines, leading to the destruction of BBB.^412,413^ These are all new insights that PRRs are involved in the body’s inflammatory response and may have an impact on the treatment of diseases.

### Endocrine diseases

In order to adapt to the constantly changing internal and external environment and maintain the relative stability of the internal environment, the human body must rely on the cooperation and regulation of the nervous, endocrine, and immune systems,^414,415^ so that the activities of various organs and systems are coordinated, and jointly shoulder all the life phenomena of the body. The immune system is not only regulated by the other two systems but also affects them through chemical information molecules and receptors.^416,417^

In patients with autoimmune thyroid disease, the expression and activation of TLR2, 3, and 9 are significantly increased.^418,419^ In addition, it has been reported that TLR9 negatively regulates pancreatic islet development and β cell differentiation, providing new directions for diabetes prevention and treatment strategies.^420^ In diet-induced obesity, TLR2 and TLR4 inhibit the replication of β cells and affect the nuclear abundance of the cell cycle regulators cyclin D2 and Cdk4. Therefore, targeting TLR2–TLR4 may alleviate the failure of β cells in diabetic patients.^421^

One of the most common complications of diabetes is diabetic foot ulcers, and the reason why it is not easy to get better is the inflammation of the wound.^422,423^ Singh et al. believe that changes in the expression level of intracellular TLRs may be one of the reasons for the continuous inflammation of chronic wounds, such as diabetic foot ulcers, and may hinder wound healing in patients with type 2 diabetes mellitus (T2DM).^424^ The basis of these pro-inflammatory effects is that intracellular TLRs activate dendritic cells and B cells to produce IFN I and III, aggravating the inflammatory response.^425–427^ The inflammatory phase of the wound healing cascade is the decisive stage of wound development, and studies have found that certain members of innate immunity play an important role in the pathogenesis of chronic wound healing abnormalities.^428,429^ The signaling pathway of intracellular TLRs is shown to be involved in some chronic inflammatory diseases, such as systemic lupus erythematosus (SLE), multiple sclerosis, hepatitis, and T2DM.^430^

### Skeletal diseases

The expression level of NOD2 in human osteoarthritis (OA) cartilage is significantly higher than that of normal cartilage, and its combined action with TLR2 contributes to the pro-catabolic gene expression induced by 29-kDa amino terminal (matrix degradation product in synovial fluid of patients with OA) in human chondrocytes.^431,432^ This indicates that the NOD2 and TLR2 cross-regulatory pathway may be a target to prevent the development of arthritis. More and more evidences indicate that the interaction between different signal pathways may be a new way for the occurrence and development of diseases. The latest research combines three biological processes in skeletal muscle during exercise, innate immune response, autophagy protein homeostasis, and adenosine monophosphate-activated protein kinase (AMPK) activation, revealing that TLR9 can regulate the energy metabolism of skeletal muscle during exercise, and TLR9 regulates exercise-induced skeletal muscle AMPK activation by interacting with the core autophagy protein beclin1.^433,434^

## Clinical therapy of PRRs

Based on the important role that PRRs play in innate immunity, they have received extensive attention in the fields of immunology and drug research.^310,435^ There are many types of PRRs and a wide range of ligands. They can be used as drug targets for tumor, inflammation, autoimmune disease, pathogenic microbial infection, and other diseases, which are important entry points for immunotherapy.^314^ The activation of PRRs brings double-sided effects: on the one hand, it stimulates innate immunity and adaptive immunity to resist pathogenic microorganisms; on the other hand, it promotes the expression of a large number of cytokines, forms an inflammatory microenvironment, and causes tissue damage.^29,436^ Therefore, the treatment strategy targeting PRRs is mainly to use ligand analogs to activate PRRs,^435^ use antagonists to inhibit their activation, or use antibodies and small molecules to inhibit PRR signaling pathways.^437^ It is reported that most of the agonists and antagonists of TLRs are only in the clinical development stage, which are studied comprehensively in PRRs (Table 2). In addition, microRNA (miRNA), exosomes, and combination therapy also have certain potential in this field.^438,439^

**Table 2:** Clinical trials investigating the use of TLR agonists and antagonists in diseases

Drug	Phase	Target	Application	Treatment	NCT number	Status
Agonists						
MGN1703	II	TLR9	Human immunodeficiency virus type 1 (HIV-1)	Monotherapy	NCT02443935	Completed
gp100	II	TLRs	Melanoma	Combination with resiquimod (R848)	NCT00960752	Completed
MAGE-3	II	TLRs	Melanoma	Combination with resiquimod (R848)	NCT00960752	Completed
Insulin	II	TLRs	Insulin resistance	Monotherapy	NCT01151605	Unknown
EMD 1201081	II	TLR9	Squamous cell carcinoma of the head and neck cancer	Combination with cetuximab	NCT01040832	Completed
Resiquimod	I	TLR7/8	Influenza vaccination in seniors	Monotherapy	NCT01737580	Completed
CPG 7909	II	TLR9	HIV infections	TLR-9 adjuvanted pneumococcal	NCT00562939	Completed
DSP-0509	II	TLR7	Advanced solid tumors	Monotherapy and combination with Pembrolizumab	NCT03416335	Recruiting
SD-101	I	TLR9	Chronic hepatitis C	Monotherapy and combination with ribavirin	NCT00823862	Completed
Imiquimod	II	TLR7	Breast cancer (for chest wall recurrences or metastases to the skin), breast neoplasms	Monotherapy	NCT00899574	Completed
Imiquimod	II	TLR7	Breast cancer, metastatic breast cancer, recurrent breast cancer	Monotherapy and combination with cyclophosphamide (CTX) and radiotherapy (RT)	NCT01421017	Completed
GSK1795091	I	TLR4	Cancer	Monotherapy	NCT02798978	Completed
GSK2245035	II	TLR7	Mild asthma and allergic rhinitis	Monotherapy	NCT01788813	Completed
SD-101	I	TLR9	Metastatic pancreatic adenocarcinoma, refractory pancreatic adenocarcinoma, stage IV pancreatic cancer, AJCC v8	Combination with nivolumab and radiation therapy	NCT04050085	Recruiting
Motolimod	II	TLR8	Ovarian cancer	Chemoimmunotherapy with anti-PD-L1 antibody MEDI4736	NCT02431559	Active, not recruiting
VTX-2337	I	TLR8	Locally advanced, recurrent, or metastatic squamous cell cancer of the head and neck (SCCHN)	Combination with cetuximab	NCT01334177	Completed
CPG 7909	II	TLR9	Non-Hodgkin lymphoma, mycosis fungoides	Monotherapy	NCT00185965	Completed
PolyICLC	I/II	TLR3	Melanoma	Adjuvants	NCT04364230	Recruiting
Antagonist						
IMO 8400	II	TLR7, TLR8, TLR9	Plaque psoriasis	Monotherapy	NCT01899729	Completed
OPN-305	II	TLR2	Delayed graft function	Monotherapy	NCT01794663	Completed
Hydroxychloroquine	III	TLR7, TLR9	Autoimmune diseases, Sjogren’s syndrome, dry eye	Monotherapy	NCT01601028	Completed
Eritoran	II	TLR4	Insulin sensitivity	Monotherapy	NCT02321111	Completed

Limited clinical studies have been carried out investigating PRR agonists and antagonists in related research to date. Most of the agonists and antagonists of TLRs are only in clinical development stage, which are studied comprehensively in PRRs

### PRR agonists for therapy

Imiquimod is the first targeted drug for TLRs. It is a specific agonist of the TLR7 receptor. It can induce the production of IFN-α, IL-6, and TNF-α to achieve the purpose of regulating immunity and treating tumors.^440^ Selgantolimod, a novel TLR8 agonist, can activate TLR8 and elicit cytokine responses in patients with chronic hepatitis B infection.^441,442^ Further studies with longer durations of selgantolimod treatment are required to evaluate efficacy. The ligand of TLR9 is unmethylated DNA, and CpG oligodinucleotide (CpG ODN) that mimics this structure has good antitumor potential (e.g., IMO-2055, MGN-1703, MGN-1704).^443^ The research of MGN-1703 on the treatment of advanced CRC is in phase III. MGN-1703 can induce a potent type I IFN response in the intestine, which is related to the subtle changes of intestinal flora.^444^ Clinically, PRR agonists can be potentially used not only as therapeutic agents to treat but also as adjuvants in conjunction with other immunotherapies.^445,446^ Due to the instability and drug resistance of existing drugs, some adjuvants targeting PRRs emerge as the times require. A TLR3-specific adjuvant, ARNAX, can enhance DC priming and CTL proliferation without cytokine toxicity.^447^ And the conjugated STING and TLR1/2 agonist Pam_3_CSK_4_-CDG^SF^ can be used as an effective adjuvant for constructing vaccines to enhance antitumor immunotherapy.^448^ NLR agonists can promote the processing and secretion of IL-1β, which is very important for activating many immune cells.^449,450^ Therefore, NLR agonists can be served as effective components of vaccines and immune stimulants.

The class II major histocompatibility complex transactivator (CIITA) is a single member of the NLRA subfamily.^451^ As the main regulator of major histocompatibility complex II, its own modification is very important in the occurrence and development of tumors. Both the deacetylation of histones and the demethylation of DNA will downregulate the transcription of CIITA.^452^ Decitabine (5-aza-2’-deoxycytidine), Entinostat (MS-275), and Trichostatin A target CIITA to promote its expression recovery in tumors. In addition, andrographolide, arglabin, formononetin, mangiferin, and other botanicals can be used to treat tumors by inhibiting NLRP3 inflammasome.^453–457^ Representative NLR agonists like Mifamurtide have been approved for clinical trials of non-metastatic sarcoma.

RLRs are involved in the recognition of viral infection by the innate immune system, and their agonists include poly I:C.^99^ Studies have revealed the link between energy metabolism and innate immunity, indicating that lactate may act as a natural inhibitor of RLR signal transduction by targeting the adaptor protein MAVS.^458^ The latest research shows that activating RIG-I before diffusing alpha-emitting radiation therapy for metastatic and low immunogenic tumors is a more effective combination therapy technique that can inhibit tumor growth and metastasis.^459^

### PRR antagonists for therapy

PRR antagonists belong to immunosuppressants, which are mainly used to inhibit the function of receptors and block the connection between receptors and ligands.^460^ They can be used to treat diseases with abnormal activation of the immune system. The abnormal activation of TLR7, 8, and 9 contributes to the onset and maintenance of inflammatory diseases.^461,462^ The inhibitors IMO-3100 and IMO-8400 targeting them can significantly reduce the expression of IL-17A induced by IL-23, which has a potential role in the inflammatory cascade.^463,464^ In addition, inhibition of TLR7 and TLR9 with IRS-954 or chloroquine may be a new treatment for HCC.^465^ In addition to the immune inhibitory oligonucleotides that can be used as TLR inhibitors, CPG-52364, a derivative of the small molecular weight chemical compound quinazoline, can also block ligand-induced activation of TLR7, 8, and 9.^462^ FP7 is a synthetic glycolipid, which can be used as TLR4 inhibitor to treat septic shock.^466^ Studies have found that inhibitory ODN-containing phosphorothioate modifications of the backbone have potential therapeutic effects for SLE and rheumatoid arthritis. ODN1411 directly binds to the extracellular domain of TLR8 and competitively inhibits its signal transduction.^467^

In the NLR family, the NLRP3 inflammasome is closely related to the pathophysiology of a variety of inflammatory diseases, so it is usually used as an inhibitor target.^298,304,305^ MCC950 is a specific NLRP3 antagonist, which can prevent its activation or maintain its activation state.^468^ β-Carotene has been shown to directly bind to the PYD of NLRP3, thereby blocking the association between NLRP3 and its adaptor protein and ultimately inhibiting the activation of NLRP3 inflammasomes.^469^ This result further complements the new pharmacological strategy to prevent NLRP3 inflammasome-driven gouty arthritis. The development of small molecule inhibitors for NLRP3 has been in full swing.^470,471^ Dai et al. reported a series of tetrahydroquinoline inhibitors. In further studies in vitro, it was found that compound 6 directly binds to the NACHT domain of NLRP3 but not to the PYD or LRR domains, which inhibits the assembly and activation of NLRP3 inflammasomes. In vivo, compound 6 can significantly inhibit the expression of IL-1β and alleviate the symptoms of colitis.^472^ Agarwal et al. identified alkenyl sulfonylurea derivatives as a novel, oral, and biologically effective NLRP3 inhibitor. Compound 7 of alkenyl sulfonylurea derivatives has good pharmacokinetic characteristics and high oral bioavailability.^473^

### Other potential therapies via PRRs

Emerging evidence suggests that the interaction between miRNA and PRR pathway and immunoregulation are intimately interwoven.^438^ The interaction between the two is particularly significant in the pathogenesis of rheumatoid arthritis (RA).^474,475^ RA is a common autoimmune disease, which is characterized by synovial hyperplasia and irreversible bone destruction caused by chronic synovitis. Rheumatoid arthritis synovial fibroblast (RASF) plays a central role in this process.^476–478^ The expression of miR-146a and miR-155 is upregulated in RASF. miR-146a inhibits the expression of TRAF-6 and IRAK-1 in the TLR signaling pathway, thereby inhibiting the production of key adaptor molecules downstream of TLRs in RA.^479^ And miR-155 negatively regulates the activation of TLRs/IL-1R inflammatory pathway. Both of them will eventually reduce RA inflammation.^480^ In addition, miR19a/b, miR-20a, and miR-10a are downregulated in RASF. Downregulated miR19a/b directly targets TLR2 to increase its expression^481^; miR-20a inhibits the expression of ASK1, a key component of the TLR4 pathway^482^; miR-10a targets IRAK4 and TAK1 to accelerate IκB degradation and NF-κB activation.^483^ These effects promote the inflammatory response of RA. All these evidences indicate that miRNAs can surpass their conventional functions and act as physiological ligands for TLRs, thereby regulating the expression of cytokines in the inflammatory response. miRNA can also target other PRRs. miR-485 can target RIG-I mRNA for degradation, leading to enhancement of virus replication and inhibition of antiviral response.^484^ miRNA-155 can aggravate the brain tissue inflammation in neonatal rats with hypoxia and ischemia by regulating the NOD1/NF-κB signaling pathway, thereby causing hypoxia and ischemic brain damage in neonatal rats.^485,486^

In addition, exosomes are also involved in the complex immune regulatory network of miRNA and PRRs.^487^ Exosomes are small membrane vesicles containing complex RNA and protein. A variety of cells can secrete exosomes under normal and pathological conditions.^488,489^ There is evidence that exosomes can regulate innate immunity by combining with TLRs, which provides a new method for the body to resist pathogens and treat diseases.^487^ Brain exosomes are rich in amyloid-beta (Aβ), the main component of neuritic plaques, which can activate TLR2, TLR4, and TLR9 signaling, thus alleviating the early symptoms of Alzheimer’s disease.^490^ It was found that the expression of miR-216a-5p in the plasma of patients with syphilis is negatively correlated with the expression of inflammatory cytokines. Exosomes containing miR-216a-5p inhibit the inflammatory response induced by recombinant Tp17 by targeting the TLR4–MyD88 pathway.^491^ Melphalan, a genotoxic agent, can enhance the ability of multiple myeloma (MM) cells to release exosomes in MM. Hsp70 on the surface of MM-derived exosomes activates TLR2 in NK cells, thereby inducing the production of INFγ.^492^ This enlightens us that exosomes with significant exposure to DAMPs can link chemotherapy with antitumor innate immune response.

However, whether they are therapeutic agents, adjuvants, or others, it still needs a long time to further improve the research in vitro and in vivo before clinical application in tumor treatment.^493^

## Conclusions and future perspectives

The innate immune system has multiple recognition mechanisms in different cell types with different subcellular structures. Recently, great progress has been made in the study of the interaction between PRRs and ligands. A preliminary understanding of the crystal structure of some PRRs and the commonalities and differences in the structural composition of different types of PRRs has been made. We understand the existing forms of PRRs alone in solution and after binding to ligands, as well as the molecules involved in the process of PRR identification and ligand binding. As the initiation step of the innate immune response and antiviral response, PRR recognition and binding of ligands is critical. PRRs recognize targets through their respective ligand-recognition domains: LRRs, CTDs, CTLDs, and a variety of DNA- or RNA-binding domains. Ligand binding induces activation and transmission of the signal, which is transmitted through cascade amplification by their respective domains—TIR domain, protein interaction domain, CARD, ITAM, and PYD. In some cases, one kind of PRRs can recognize multiple PAMPs, and one kind of PAMPs can also be recognized by different PRRs, which can induce an inflammatory response and adaptive immune response in a synergistic manner.^494^

PRRs that are difficult to find and identify and complex ligand-recognition mechanisms have hindered the study of the role of the innate immune system in related diseases and even tumors. In previous studies, computational modeling and mutational analysis of human TLR10 showed that the structure of TLR10 was modeled with the explicit inclusion of TLR2 and lipopeptide structures.^495^ Recently, RRGPredictor, a tool mainly based on text mining and set theory to predict new plant PRRs, makes the identification of PRRs more effective, specific, and sensitive than other available tools.^496^ And a random forest-based method was proposed to identify PRRs, which is superior to other machine learning methods for PRRs. This method constructs a benchmark database, uses the amino acid composition and the composition transition distribution to formulate the sequences in the dataset, and then uses the maximum relevance maximum distance to select the best features. However, due to the small amount of data and the low sensitivity of the method, the identification results for PRRs are still unsatisfactory.^497^ Therefore, the ability to develop a vertebrate PRR predictor based on protein domain similarity and homology modeling is becoming an important part of this story. In addition, the future studies on the how membrane PRRs are secreted into body fluids and act as soluble PRRs will undoubtedly further our understandings of their roles in antimicrobial immune defense and autoimmune and autoinflammatory disorders. Therefore, the study of PRR-mediated pattern recognition mechanisms will help to elucidate the signaling pathways and mechanisms of disease, provide new targets and methods for the treatment of diseases, and promote the development of immunology and oncology research and improvements in their theoretical systems.
